# Supplementary figures and images for: 3D culture of functional human iPSC-derived hepatocytes using a core-shell microfiber
Source: PLoS One. 2020 Jun 11;15(6):e0234441. doi: 10.1371/journal.pone.0234441 (PMC7289419; doi:10.1371/journal.pone.0234441)

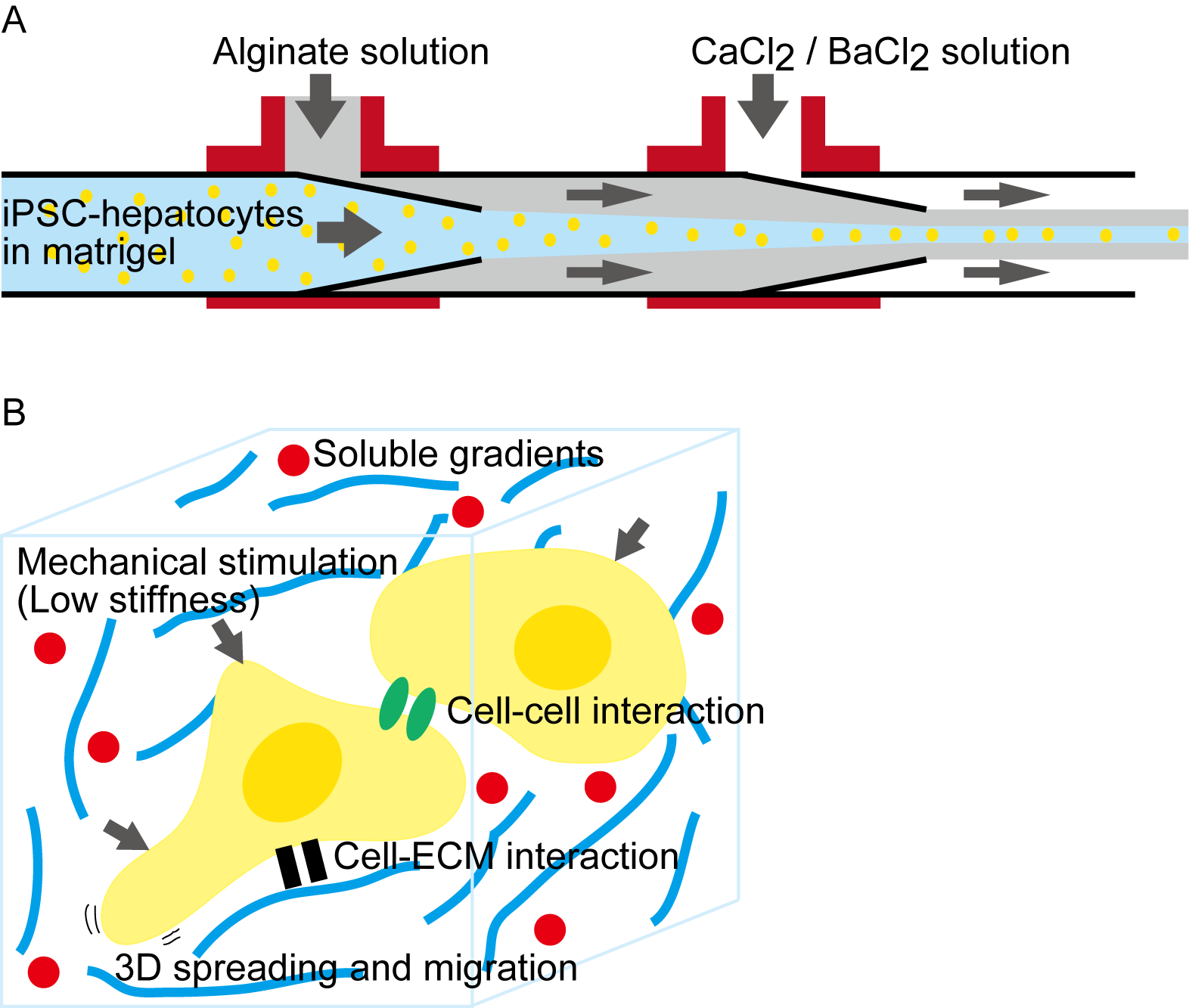

Supplement: S1 Fig — (A) A double co-axial laminar flow microfluidic device was used for the formation of the iPSC-hepatocyte-laden core-shell hydrogel microfiber (iPSC-hepatocyte fiber). (B) The cells interacted with both other cells and the ECM, and were mechanically stimulated and regulated to spread and migrate three-dimensionally. Gradients of soluble factors, nutrients, and oxygen are also generated by diffusion through the ECM gel. (TIF) [file pone.0234441.s001.tif]

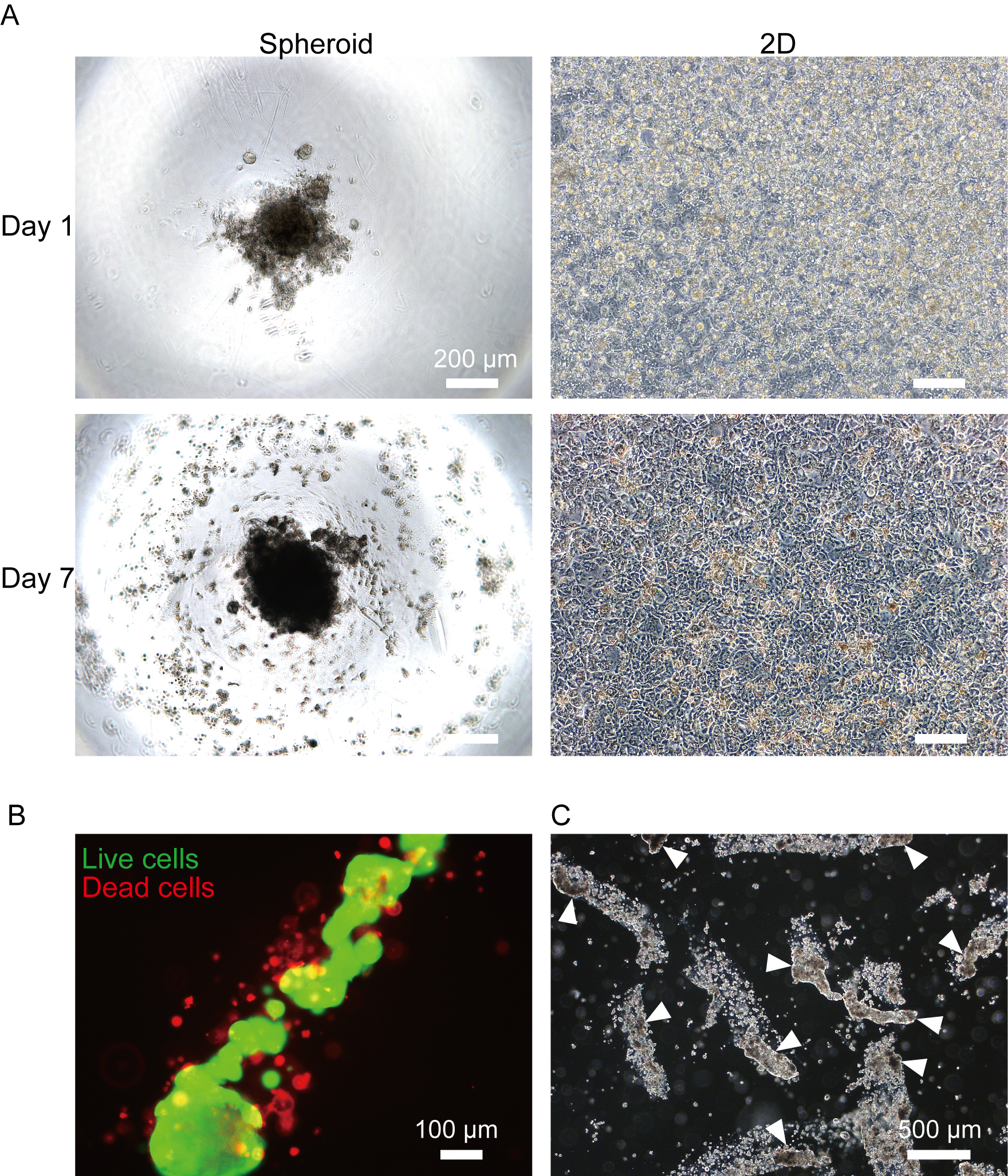

Supplement: S2 Fig — (A) Human iPSC-hepatocytes formed loose and distorted spheroid in the suspension culture and exhibited a cobblestone morphology in the 2D culture on Matrigel-coated plate. (B) Live/dead staining was performed 7 days after the 3D culture of the hepatocyte fibers. The iPSC-hepatocytes in the cell aggregates were alive. (C) The shell of the fibers was degraded by alginate lyase after the 3D culture. The iPSC-hepatocytes maintained their compacted cell aggregates (arrowheads). (TIF) [file pone.0234441.s002.tif]

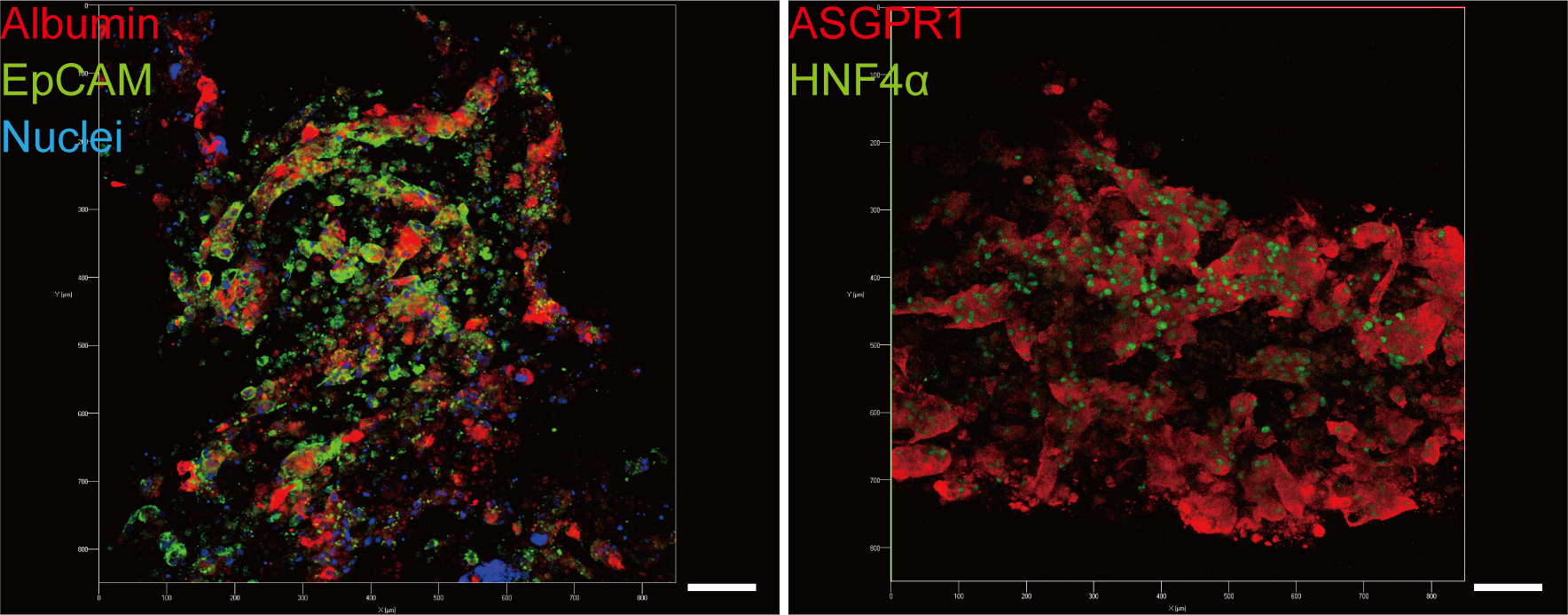

Supplement: S3 Fig — Immunocytochemistry was performed for the hepatic stem/progenitor marker EpCAM, and the hepatic marker HNF4α. Some of the iPSC-hepatocytes were positive for EpCAM merging with albumin, and almost of HNF4α-positive cells were also positive for ASGPR1. (TIF) [file pone.0234441.s003.tif]

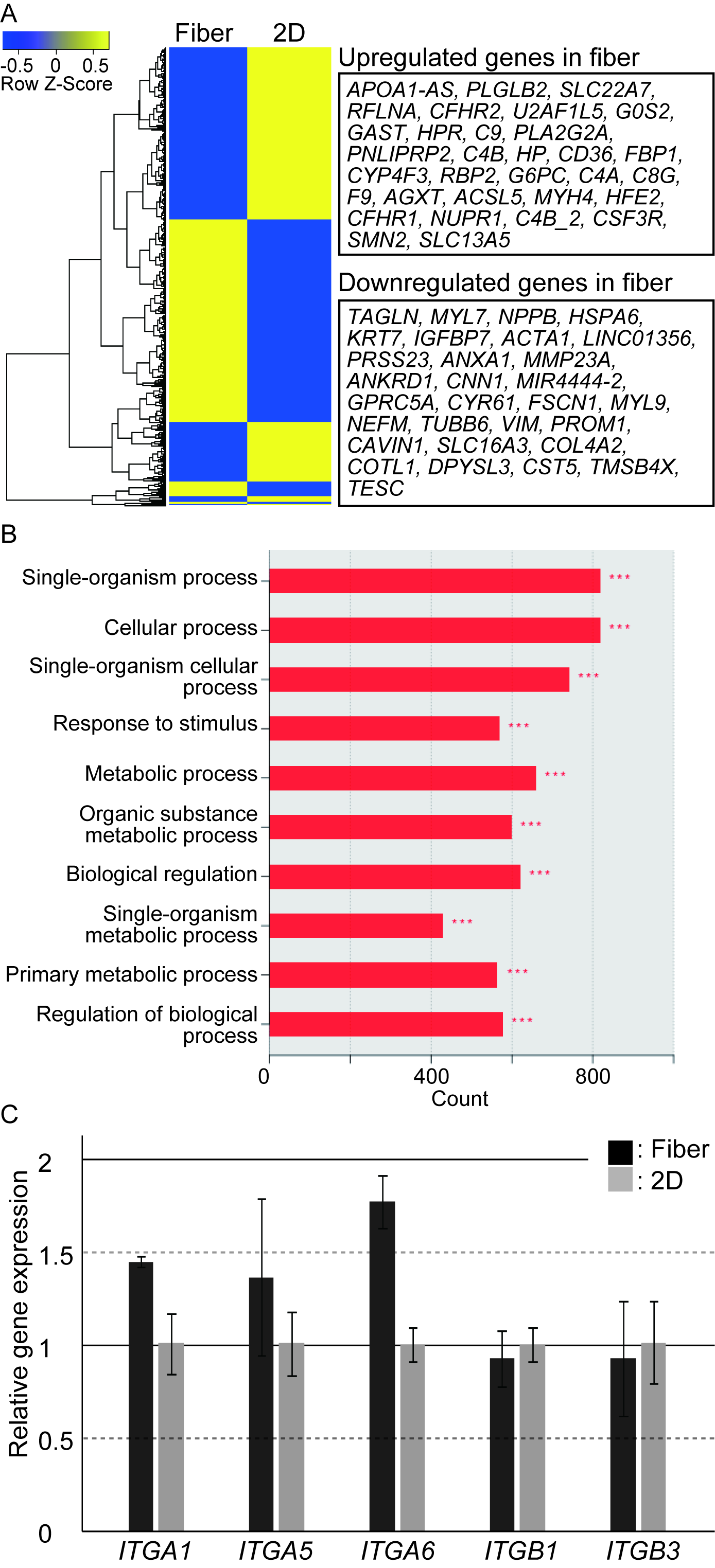

Supplement: S4 Fig — (A) RNA-sequencing was performed for hierarchical clustering analysis. Gene expression level is shown in normalized value (log2 based) using z-score, and color-coded with the color range shown in the top. Top 30 genes upregulated and downregulated in the fiber were selected and indicated. (B) Gene-set enrichment analysis which based on GO was conducted with the significant gene list and progressed about 3 categories of GO (biological processes, cellular component, and molecular function). The bar plot shown here is the top 10 terms of GO functional analysis in biological processes (*; P<0.05), **; P<0.01, ***; P<0.001). (C) qRT-PCR was performed for quantifying the expression of the integrin genes (n = 3). The error bars represent the s.d. of triplicate samples. (TIF) [file pone.0234441.s004.tif]

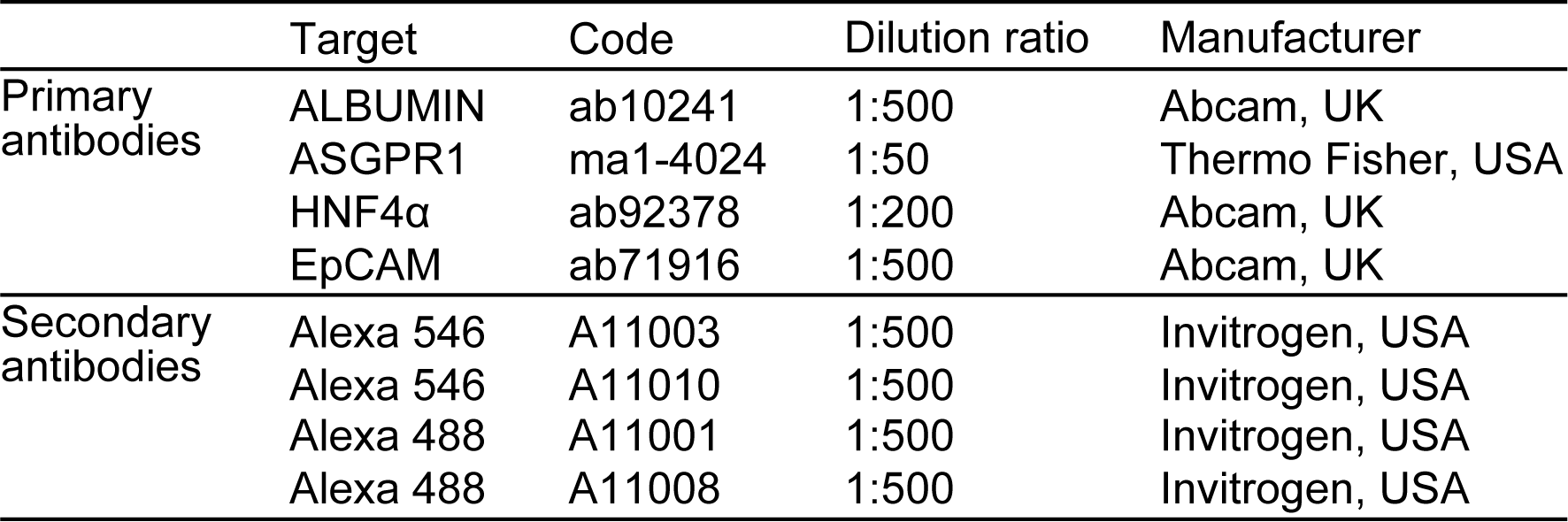

Supplement: S1 Table — (TIF) [file pone.0234441.s005.tif]

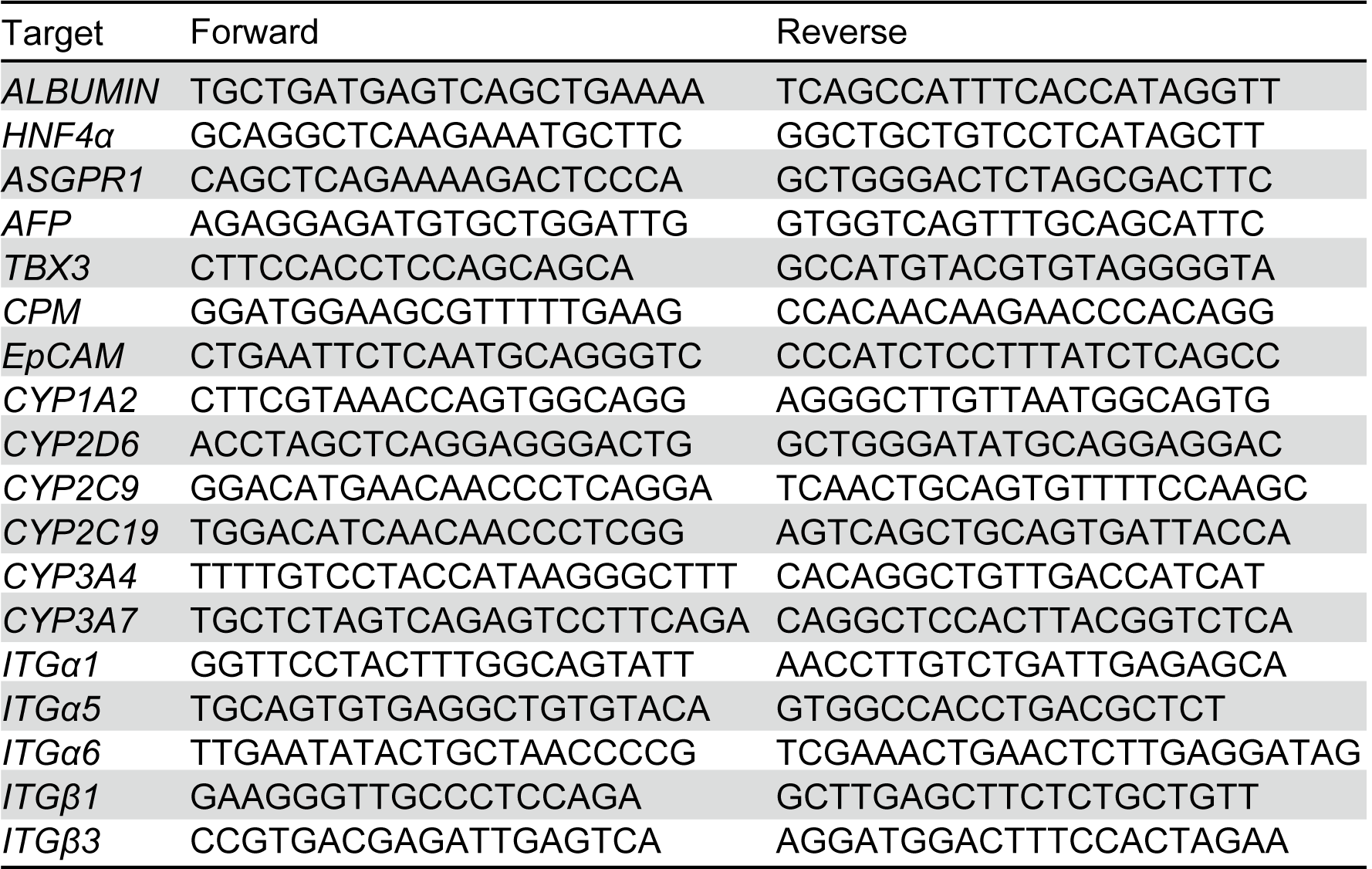

Supplement: S2 Table — (TIF) [file pone.0234441.s006.tif]
